# Incidence, Outcomes, and Risk Factors for Isoniazid-Resistant Tuberculosis from 2012 to 2022 in Eastern China

**DOI:** 10.3390/antibiotics13040378

**Published:** 2024-04-22

**Authors:** Yan Shao, Wenlei Song, Honghuan Song, Guoli Li, Limei Zhu, Qiao Liu, Cheng Chen

**Affiliations:** 1 Center for Disease Control and Prevention of Jiangsu Province, Department of Chronic Communicable Disease, Nanjing 210009, China; shaoyan@jscdc.cn (Y.S.); songhh@jscdc.cn (H.S.); ligl@jscdc.cn (G.L.); zhulimei@jscdc.cn (L.Z.); liuqiao@jscdc.cn (Q.L.); 2 Center for Disease Control and Prevention of Kunshan, Suzhou 215300, China; wenleisong@163.com

**Keywords:** tuberculosis, isoniazid-resistant TB, treatment outcome, fluoroquinolone

## Abstract

Background: Isoniazid-resistant, rifampicin-susceptible tuberculosis (Hr-TB) is the most frequent drug-resistant tuberculosis (DR-TB) in the world, and unfavorable outcomes of Hr-TB are more common compared to drug-susceptible TB. Considering there is no optimal regimen accepted worldwide, we undertook a retrospective cohort study in eastern China to estimate incidence trends and risk factors associated with unfavorable outcomes of Hr-TB. Methods: Between January 2012 and December 2022, all Hr-TB patients’ information was extracted from the Tuberculosis Information Management System (TIMS), which is a national electronic information platform, to record TB patients’ clinical information in this study. The incidence of Hr-TB was determined by the mid-year population according to census data published by the government. We categorized treatment regimens depending on fluoroquinolone (FQ) use, and potential risk factors were analyzed using multivariable logistic regression. Results: A total of 3116 Hr-TB patients fulfilled the inclusion criteria and were enrolled in this study. The average annual rate of Hr-TB in the 11 years under investigation was 0.34 per 100,000 and increased to 0.53 per 100,000 until 2019. In total, six different treatment regimens were utilized in the study sites, and less than 1% of regimens adopted FQ. There was no difference in the unfavorable outcomes between the FQ-included and FQ-excluded groups (*p* = 0.22). The average treatment duration was 7.06 months, and the longest treatment was 26 months. Approximately 20% (637/3116) of Hr-TB patients had unfavorable outcomes, and 60.13% (383/637) of them proceeded to multidrug-resistant tuberculosis (MDR-TB). Treatment duration and a positive smear at the end of the 5th month were significantly associated with unfavorable outcomes (*p* < 0.001). Conclusion: The unfavorable treatment outcomes of Hr-TB are still high in eastern China, and the efficacy of FQ-containing regimens needs to be validated for Hr-TB treatment.

## 1. Introduction

Tuberculosis (TB) remains one of the world’s leading causes of death from a single infectious agent, and it was estimated that about 10.6 million people were newly diagnosed as TB patients in 2022 [1]. Meanwhile, the drug resistance of *Mycobacterium tuberculosis* (Mtb) is a main obstacle to global TB control. According to the World Health Organization (WHO), there were 3.3% and 17% estimated proportions of multidrug-resistant/rifampicin-resistant tuberculosis (MDR/RR-TB) among new cases and previously treated cases, respectively [2]. Compared to drug-susceptible TB patients, the treatment of drug-resistant TB (DR-TB) requires longer duration and more highly toxic medicines with worse outcomes [3].

Until now, the most common form of DR-TB is isoniazid-resistant, rifampicin-susceptible tuberculosis (Hr-TB), which is raising concern globally [4]. Isoniazid (H) is an essential first-line anti-TB drug for both active and latent TB, with benefits including its early bactericidal activity and good safety profile. Furthermore, the inappropriate treatment of unidentified Hr-TB can generate further resistance to the other anti-TB agent, rifampin, and result in MDR. The average proportions for Hr-TB were 7.1% and 7.9% in new TB cases and previously treated TB cases, respectively, according to an assessment conducted in 149 countries from 2003 to 2017 [5]. Recently, Hr-TB rates surged to 13.1% among new cases and 17.4% among previously treated cases in 2019 [6]. A recent systematic review revealed that Hr-TB patients receiving the WHO standard regimen for new TB cases had worse treatment outcomes, such as higher treatment failure (11% vs. 1%), more relapses (10% vs. 5%), and higher acquired drug resistance rates (8% vs. 0.3%) [7]. To address the overall growing problem of Hr-TB, the WHO conducted an individual patient data (IPD) analysis to identify the optimal treatment regimen for Hr-TB and recommended a 6REZ-Lfx regimen (6 months of levofloxacin (Lfx), rifampicin (R), pyrazinamide (Z), and ethambutol (E)) without streptomycin (S) or any other injectable agents. However, this conditional recommendation has very limited evidence for the estimates of its effects according to the GRADE methodology, and the necessity of fluoroquinolone and the treatment duration is under debate [8].

In China, the first national drug resistance survey conducted during 2006–2007 estimated that the rate of Hr-TB was approximately 16% and 38.5% among new and previously treated TB cases, respectively [9]. Furthermore, the proportion of INH resistance increased significantly from 2005 to 2015 from 30% to 39% according to research in Beijing [10]. We also assessed the Hr-TB burden and treatment outcomes in Jiangsu province, where 4.6% were Hr-TB patients identified employing a phenotypic drug-susceptibility test (DST) and among them, 17.5% had unfavorable outcomes [11]. It is well known that drug-resistant tuberculosis represents a growing threat to public health and economic growth. A previous study conducted in eastern China indicated that medical and pretreatment costs lead to high costs of TB care, especially among patients from the poorest households [12].

Although molecular testing is now applied widely in eastern China, the isoniazid-resistance profile is not prioritized for diagnosis according to the National TB Program (NTP) and is only carried out for cases with unfavorable treatment outcomes. Therefore, Hr-TB patients often receive the first-line anti-TB drugs. Additionally, the treatment regimen could also be individualized by the clinicians according to the patient’s condition, adverse reactions, or treatment adherence. There was a paucity of data to assess the clinical treatment and management of Hr-TB. Therefore, we evaluated the epidemiology trend and risk factors for Hr-TB with poor outcomes in the period 2011–2022 in the eastern part of China and provide evidence for improving the clinical treatment and management of Hr-TB.

## 2. Results

### 2.1. Trend and Distribution of Hr-TB Cases

**Figure 1:**
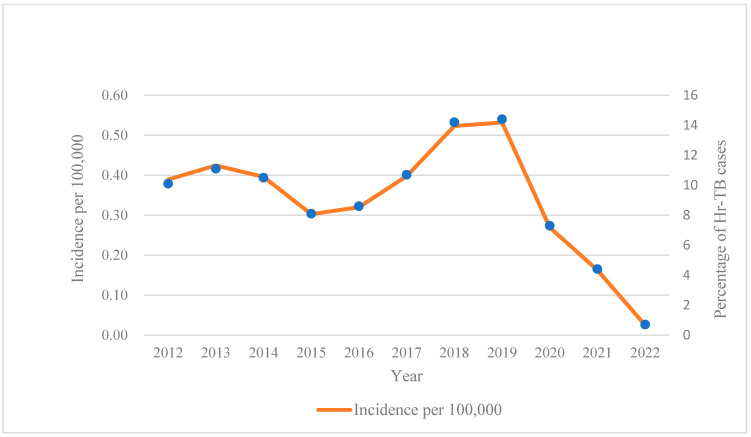
Annual incidence of Hr-TB per 100,000 during 2012–2022 in Jiangsu province. Abbreviation: Hr-TB, isoniazid-resistant, rifampicin-susceptible tuberculosis.

During the study period, 3237 Hr-TB patients were enrolled, and 121 lacked the necessary information (treatment regimens or outcomes). After excluding such ineligible participants, 3116 Hr-TB patients were enrolled for analysis. Among them, 1325 were detected by the phenotypical DST method, and 1791 were detected by molecular DST testing. The median age of the participants was 54 years old (IQR:35–67), and 77.60% were male. To assess the trend of Hr-TB in the past 11 years, we calculated the incidence of Hr-TB based on the mid-year population according to the census data published by the government. (Figure 1) The average annual incidence rate was 0.34/100,000 and fluctuated from 2011 to 2022, obviously. After a slight decline in 2015, it steadily rose to a peak of 0.53/100,000 in 2019, then took a sudden turn and decreased rapidly in 2020. 

**Figure 2:**
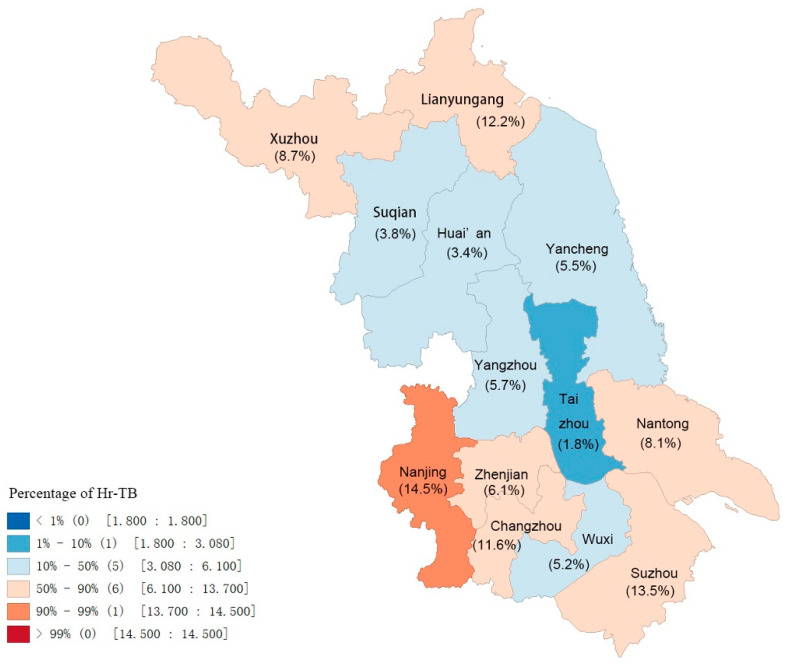
Distribution of Hr-TB in Jiangsu province depending on percentage. Abbreviation: Hr-TB, isoniazid-resistant, rifampicin-susceptible tuberculosis.

The distribution of Hr-TB in the whole province is demonstrated in Figure 2; all 13 cities were classified into four grades depending on the case percentage, which varied among different regions. Taizhou City, located in the middle of the province, had the lowest case percentage (1.77%), and the highest was observed in Nanjing City (14.52%). The central part of Jiangsu province seemed to have a relatively lower proportion of Hr-TB compared to the southern and northern parts.

### 2.2. Treatment Regimen and Outcome

Six different sets of treatment reagents were adopted during the study period and classified into two groups depending on fluoroquinolone (FQ) use (Table 1). Among them, 2105 patients (2105/3116, 67.55%) were treated with 2HRZE/4HR, which was recommended by the WHO for new patients with susceptible TB [13]. In this study, the 2HRZE/7-10HRE and 2HRZE/10HRE regimens accounted for 10.08% (314/3116) and 1.16% (36/3116) respectively. A total of 641 (641/3116, 20.57%) patients received an alternative individual regimen without FQ. Only 20 (20/3116, 0.64%) cases initiated a regimen with FQ; among them, 3 (3/20, 15%) patients received 6-9RZELfx, a standard regimen for Hr-TB according to the WHO [14]. The remaining 17 cases received individualized regimens due to severe complications or an adverse reaction. The average duration of therapy was 7.06 months, and the longest was 26 months. A total of 22 cases were treated for less than one month; 13 (65.00%) of them were amended diagnoses from Hr-TB into MDR-TB, and 8 died from TB or other reasons. Only one TB case terminated therapy because of adverse effects. Overall, 2479 (79.56%) patients were cured or completed the treatment, and unfavorable outcomes occurred in 637 (20.44%) patients. Most of those with unfavorable outcomes (383/637, 60.13%) developed MDR-TB during the treatment period. Although patients receiving regimens with FQ had the lowest rate of unfavorable outcomes (15%), there was no significant difference among the different regimen subgroups (*p* = 0.22) (Table 2).

### 2.3. Risk Factors for Unfavorable Outcomes of Hr-TB

Two general demographic factors (age and gender) and another four clinical factors (patient delay, treatment duration, and smear results at the end of second and fifth months) were involved in univariate and multivariate logistic regression. The associations between the risk factors and unfavorable outcomes are shown in Table 3 and Table 4.

Compared to females, male patients were more likely to suffer unfavorable outcomes, although there was no significant difference (*p* > 0.05). The risk for unfavorable outcomes increased with age, but there was still not a statistical difference in this study (*p* > 0.05). Moreover, compared to the standard 6-month treatment duration, prolonged therapy, despite the drug combination, could improve outcomes prominently (*p* < 0.001). Noticeably, a subgroup of patient delay from 2 weeks to 1 month demonstrated a faint advantage compared to the subgroup of less than two weeks (adjusted OR = 0.88, 95% CI = 0.68–1.08, *p* = 1.19). A similar result was found in the group of the second month smear; the negative subgroup had a lower risk related to unfavorable outcomes (adjusted OR = 1.08, 95% CI = 0.79–1.47, *p* = 0.64). Furthermore, a positive smear at the end of the fifth month was a significant risk factor, with an odds ratio of 66.97 (95% CI = 38.26–117.22, *p* < 0.01). Meanwhile, longer treatment and negative smear results at the end of the 5th month were both protective factors with a significant differences in the multivariable model (*p* < 0.001).

## 3. Materials and Methods

### 3.1. Study Population

In total, the 13 cities and 68 counties in Jiangsu province each have designated tuberculosis hospitals to provide local medical services to TB patients. Usually, those who suspect TB access the health service at the county level hospital for TB diagnosis, and the municipal hospital provides DST, including rifampicin, isoniazid, and other anti-TB drugs. 

We conducted a retrospective cohort study and enrolled all confirmed isoniazid-resistant, rifampicin-susceptible tuberculosis (Hr-TB) patients based on either phenotypic or molecular DST from the whole province, from January 2012 to December 2022. TB-designed hospitals provide phenotypic DST for TB patients with at least four kinds of anti-TB drugs (rifampin, isoniazid, amikacin, and ofloxacin), and at least two kinds of anti-TB drugs for the molecular method (rifampin and isoniazid). The patient clinical information was collected by the Tuberculosis Information Management System (TIMS). This system belongs to the National Infectious Disease Database and is used to record TB diagnosis, patient demographics, laboratory testing results, clinical treatment regimens, and outcomes, such as cured, completed treatment, transferred to MDR treatment, death, and loss to follow-up. Patients were excluded if they were identified as NTM (nontuberculosis mycobacterium) or if there was insufficient information on the treatment regimens and outcomes. We exported all eligible participants’ data and subsequently anonymized them by removing the names and identification numbers and replacing them with a serial number. 

### 3.2. Diagnosis Delay

Diagnosis delay was defined as the time interval between the onset of symptoms and the clinical diagnosis of active TB. Furthermore, diagnosis delay is categorized into two types: a delay in seeking medical service of the patient (patient delay), and diagnosis of TB in hospital (diagnosis delay). In this study, we only collected data on patient delay, meaning the time interval from the onset of symptoms to consultation with a doctor.

### 3.3. Treatment for Hr-TB

We collected all patients’ regimen information and categorized them by fluoroquinolone (FQ) use. If FQ was included in any regimen during the treatment period, it was classified into the FQ-included group; otherwise, it was classified into the FQ-excluded group. Additionally, the duration of treatment was also gathered for the analysis of therapeutic effect.

### 3.4. Treatment Outcomes

According to the WHO, there are seven kinds of treatment outcomes, including cured, treatment completed, treatment success, death, failure, loss to follow-up, and unevaluated [15]. Considering the TIMS provided most categories of outcomes in accordance with the WHO, except unevaluated, which was identified as “others”, we classified the treatment outcomes in this study into two groups: cured, treatment completion, and treatment success were considered favorable outcomes; the remaining outcomes, including failure, death due to any cause, loss to follow-up, adverse reaction, transfer to MDR, and others, were identified as unfavorable outcomes. 

### 3.5. Statistical Analysis

The Pearson χ^2^ test was used for categorical comparison analysis. Univariate and multivariate logistic regression were applied to evaluate the risk factors associated with the unfavorable outcomes (failure, death due to any cause, loss to follow-up, adverse reaction, and transfer to MDR combined). All tests were two-sided, and a *p*-value of <0.05 was considered statistically significant. All analyses were performed using SAS 9.3 software (SAS Institute, Inc., Cary, NC, USA).

## 4. Discussion

In the past decade, Hr-TB has been an ongoing problem, and impeded TB control in Jiangsu province. Most Hr-TB patients are treated with regimens without FQ; about 20% (637/3116) of the patients in this study had unfavorable treatment outcomes, and more than half of them developed into MDR-TB.

From 2020 to 2022, globally, there was a substantial reduction in tuberculosis detection and case notifications due to COVID-19 [16]; however, a distinct upward trend of Hr-TB incidence was observed from 2012 to 2019. Our results are consistent with a multi-center, retrospective study in Canada, where the Hr-TB incidence increased between 2006 and 2018 [17]. However, the average annual incidence rate of Hr-TB in our study was much higher than that in the Canadian study (0.35/100,000 vs. 0.25/100,000). Besides the high incidence of Hr-TB, the widespread distribution of cases is another concern. Boundary cities in Jiangsu province presented the majority of Hr-TB cases. This characteristic of Hr-TB distribution should be considered by the local health departments, and isoniazid-resistance detection needs to be performed prior to treatment in such high-incidence areas.

According to the national tuberculosis control policy in China, rifampicin resistance is considered a proxy of MDR-TB. The Xpert MTB/RIF assay (Cepheid Sunnyvale, CA, USA) has been applied in county-level hospitals; once patients are detected as rifampicin- resistant, and they are transferred to a city-level hospital and treated with an MDR-TB treatment regimen. On the other hand, patients without resistance to rifampicin are initially treated with the standard regimen comprising only first-line drugs regardless of isoniazid resistance. Second-line drugs, like fluoroquinolone, are rarely prescribed by clinicians because of the lack of isoniazid-resistance detection.

Based on this strategy, six different regimens were adopted during our study period; among them, the FQ-included regimen accounted for a very low rate, and standard drug-susceptible regimens for new cases were used for more than 60% of Hr-TB patients. A previous meta-analysis revealed that first-line drugs were insufficient for treating Hr-TB and could result in suboptimal outcomes. Furthermore, empirical treatment of Hr-TB could generate MDR-TB, particularly in settings where the prevalence of Hr-TB is high [7]. Research conducted in Africa adopted a combination of first-line drugs (2RHESZ/RHEZ/5RHE) and indicated that 29% of Hr-TB patients received unfavorable outcomes [18]. Similar results were found in our study; about 20% of Hr-TB patients treated with 2HRZE/4HR obtained unfavorable outcomes, and 70.25% (307/437) developed MDR later.

Although the WHO recommended a 6-month four-drug regimen, including rifampicin, ethambutol, pyrazinamide, and levofloxacin for the treatment of Hr-TB in 2018, there is continuing controversy regarding the necessity and effectiveness of FQ. In some studies, supplementation with fluoroquinolone has been beneficial for improving treatment success [19,20]. On the contrary, a retrospective cohort study conducted in Europe presented no significant difference in the odds of an overall negative outcome between regimens with and without fluoroquinolone [21]. Another multicenter cohort study conducted in Korea compared two regimens, 6-9REZ and 2REZ/7-10RE; they also suggested that the additional use of fluoroquinolone was not associated with favorable outcomes in the whole cohort but benefited the 2REZ/7-10RE subgroup due to a shortened pyrazinamide administration [22]. There were only 20 cases treated with a regimen that included FQ in our study, and the FQ subgroup showed a faint advantage in positive treatment outcomes (*p* = 0.22). Because of the significant heterogeneity among studies, there is currently no strongly agreed-upon standard regimen for Hr-TB patients; most of them receive inappropriate clinical treatment, which becomes the key precursor for the development of MDR-TB. Thus, Hr-TB should be taken seriously, not only with its timely detection but also its proper treatment. However, the sample size of the FQ group was too small in this study, and the optimal regimen could not be deduced from the analysis. Therefore, we recommend that future research utilizes adequate samples and appropriate comparison groups to analyze the optimal combination of anti-TB drugs for Hr-TB.

Previous studies have focused on risk factors for unfavorable outcomes of Hr-TB, showing inconsistent results regarding age and gender [23,24], neither of which showed significant differences in our study. In contrast, treatment duration and the smear results at the 5th month were significantly associated with unfavorable treatment outcomes. Previous studies revealed that positive smear results, especially at initial testing, were associated with poor outcomes [25,26]. In our study, positive smear results at the end of the 5th month were also a strong predictor of unfavorable outcomes. Additionally, we found that a longer duration of treatment was significantly associated with unfavorable outcomes compared to a standard 6-month duration regardless of the type of regimen. Similar to our result, a former study demonstrated that if the duration of (H)RZE treatment was long enough, it could lead to better treatment outcomes than a regimen with FQ [21]. Considering that the majority of regimens in our study did not contain FQ, we speculate that fluoroquinolone may not be necessary for favorable outcomes if treatment could be implemented long enough.

In China, tuberculosis testing and treatment for drug-susceptible TB are free, and the government recommends that people seek medical services for tuberculosis if they have TB symptoms for 14 days or more. Moreover, in Jiangsu province, more advantageous measures have been taken to diagnose and treat DR-TB patients, like free molecular testing of RR-TB and free second-line anti-TB drugs for MDR-TB patients, including new medicines such as linezolid, bedaquiline, and clofazimine. But there are still many challenges, for example, persistent funding, multi-level coordination within the government, and more effective patient management. Even so, this study also revealed that patient delay was frequent and lengthy in clinical settings and significantly associated with many symptoms [27]. In this study, more than half of the patients had delayed access to medical services, and patients delayed between 2 weeks and 1 month had a lower risk of suffering poor outcomes. We considered that at two weeks after symptom onset, patients might present more typical clinical features of TB, leading to accurate and efficient diagnosis. Therefore, the government should strengthen the promotion of TB science knowledge and provide more convenient testing for Hr-TB patients at the primary hospitals.

The present study has some limitations. First, fluoroquinolone was not adopted widely during the study period, resulting in very few data on FQ-included regimens being analyzed. Therefore, the optimal regimen for Hr-TB could not be determined due to insufficient samples. Second, patients’ information depends on the digital TIMS, which cannot provide comprehensive clinical details, like the treatment duration of pyrazinamide, which could also be considered a potential risk factor for unfavorable outcomes. Third, a fluoroquinolone resistance profile was not involved in this study, while the prevalence of FQ-resistant TB is relatively high in China, according to a former study [28]. We advise future work to consider this to assess the impact of FQ resistance on treatment outcomes.

## 5. Conclusions

Overall, compared to the low TB burden region, the prevalence of Hr-TB in eastern China has been relatively high in the last decade, and about 20% of Hr-TB patients suffered unfavorable outcomes. The duration of treatment and the smear results at the end of the 5th month were significantly associated with unfavorable outcomes for Hr-TB patients. Increased attention should be paid to the timely diagnosis of isoniazid resistance and the proper treatment regimen for Hr-TB patients.

## Figures and Tables

**Table 1:** Regimens utilized for Hr-TB and outcomes observed.

FQ Included	Regimen	Number (%)	Duration of Treatment	Outcomes						
				Cured	Treatment Completion	Treatment Failed	Transfer to MDR-TB	Death	Adverse Effect	Others
Yes	6-9RZELfx	3 (0.10%)	≥6 months:3	3	0	0	0	0	0	0
	Other regimen including FQ	17 (0.54%)	≤6 months:6; 7–9 months: 2; 10-12 months: 7	13	1	0	2	0	1	0
No	2HRZE/10HRE	36 (1.16%)	≤6 months:8; 7–9 months: 4; >12 months: 24	24	6	3	3	0	0	0
	2HRZE/7-10HRE	314 (10.08%)	≤6 months:45; 7–9 months:211; 10–12 months:41; >12 months: 17	255	9	14	5	19	4	8
	2HRZE/4HR	2105 (67.55%)	≤6 months:1621; 7–9 months: 314; 10–12 months: 93; >12 months: 77	1486	182	57	307	59	4	10
	Alternative individual regimen without FQ	641 (20.57%)	≤6 months:221; 7–9 months: 220; 10–12 months: 148; >12 months: 52	469	31	30	66	37	2	6

Abbreviations: Hr-TB, isoniazid-resistant, rifampicin-susceptible tuberculosis; FQ, fluoroquinolone; R, rifampicin; H, isoniazid; Z, pyrazinamide; E, ethambutol; Lfx, levofloxacin.

**Table 2:** Comparison of different treatment regimens for Hr-TB with unfavorable outcomes.

Regimen	Total (n)	Outcomes		*p* Value
		Favorable (%)	Unfavorable (%)	
Regimen including FQ	20	17(85.00%)	3(15.00%)	0.22
2HRZE/4HR	2105	1668(79.24%)	437(20.76%)	
2HRZE/7-10HRE	314	264(84.08%)	50 (15.92%)	
2HRZE/10HRE	36	30(83.33%)	6 (16.67%)	
Alternative individualized regiment without FQ	641	500 (78.00%)	141(21.00%)	

Abbreviations: Hr-TB, isoniazid-resistant, rifampicin-susceptible tuberculosis; FQ, fluoroquinolone; R, rifampicin; H, isoniazid; Z, pyrazinamide; E, ethambutol; Lfx, levofloxacin.

**Table 3:** Univariate analysis of risk factors associated with Hr-TB unfavorable outcome.

Risk Factor	No. (%)	Unfavorable Outcomes (%)	Crude OR	95% CI	*p*-Value	Adjusted OR *	95% CI	*p*-Value
Gender								
Female	698 (22.40)	130 (20.41)	1			—		
Male	2418 (77.60)	507 (79.59)	1.16	0.94–1.44	0.18	—		
Age								
≤25	399 (12.80)	84 (13.20)	1			—		
26–40	557 (17.89)	111 (17.43)	0.93	0.68–1.29	0.67	—		
41–55	676 (21.69)	135 (21.19)	0.94	0.69–1.27	0.67	—		
56–70	937 (30.07)	186 (29.20)	0.93	0.70–1.24	0.62	—		
≥71	547 (17.55)	121 (18.90)	1.07	0.78–1.46	0.69	—		
Patient delay (day)								
≤14	1311 (42.07)	276 (43.33)	1			1		
15–30	696 (22.34)	129 (20.25)	0.85	0.68–1.08	0.18	0.88	0.68–1.08	0.19
31–-90	751 (24.10)	154 (24.18)	0.97	0.78–1.21	0.77	0.97	0.78–1.22	0.82
91–180	195 (6.26)	42 (6.59)	1.03	0.71–1.49	0.88	1.04	0.72–1.51	0.82
≥181	163 (5.23)	36 (5.65)	1.06	0.72–1.58	0.76	1.09	0.73–1.61	0.69
Duration of treatment (day)								
≤180	656 (21.05)	523 (82.10)	1			1		
180–270	1791 (57.48)	82 (12.87)	0.01	0.01–0.02	<0.01	0.01	0.009–0.016	<0.001
271–360	365 (11.71)	25 (3.92)	0.02	0.01–0.03	<0.01	0.02	0.011–0.028	<0.001
≥361	304 (9.76)	7 (1.10)	0.01	0.00–0.01	<0.01	0.06	0.003–.013	<0.001
Smear results at the end of 2nd month ^$^								
Negative	2719 (90.81%)	513 (90.32%)	1					
Positive	275 (9.19%)	55 (9.68%)	1.08	0.79–1.47	0.65	1.08	0.79–1.47	0.64
Smear results at the end of 5th month ^#^								
Negative	2527 (96.56%)	150 (67.26%)	1					
Positive	90 (3.44%)	73 (32.74%)	68.05	39.14–118.30	<0.01	66.97	38.26–117.22	<0.001

* Adjusted for age and gender. ^$^ A total of 2994 cases provided smear results at the end of 2nd month. ^#^ A total of 2617 cases provided smear results at the end of 5th month. Abbreviation: Hr-TB, isoniazid-resistant, rifampicin-susceptible tuberculosis.

**Table 4:** Multivariate analysis of risk factors associated with unfavorable outcomes of Hr-TB.

Risk Factor	No. (%)	Unfavorable Outcomes (%)	OR	95% CI	*p*-Value
Gender					
Female	698 (22.40)	130 (20.41)	1		
Male	2418 (77.60)	507 (79.59)	1.21	0.77–1.86	0.4
Age					
≤25	399 (12.80)	84 (13.20)	1		
26–40	557 (17.89)	111 (17.43)	0.92	0.48–1.79	0.81
41–55	676 (21.69)	135 (21.19)	1.23	0.66–2.30	0.51
56–70	937 (30.07)	186 (29.20)	0.95	0.52–1.74	0.86
≥71	547 (17.55)	121 (18.90)	1.3	0.69–2.46	0.42
Duration of treatment (day)					
≤180	656 (21.05)	523 (82.10)	1		
180–270	1791 (57.48)	82 (12.87)	0.07	0.05–0.10	<0.001
271–360	365 (11.71)	25 (3.92)	0.08	0.05–0.14	<0.001
≥361	304 (9.76)	7 (1.10)	0.03	0.01–0.08	<0.001
Smear results at the end of 5th month *					
Negative	2527 (96.56%)	150 (67.26%)	1		
Positive	90 (3.44%)	73 (32.74%)	30.48	15.84–58.63	<0.001

* A total of 2617 cases provided smear results at the end of 5th month. Abbreviation: Hr-TB, isoniazid-resistant, rifampicin-susceptible tuberculosis.

## Data Availability

The raw data supporting the conclusions of this article will be made available by the authors upon request.
